# Cold-Sprayed Composite Metal-Fluoropolymer Coatings for Alloy Protection against Corrosion and Wear

**DOI:** 10.3390/ma16030918

**Published:** 2023-01-18

**Authors:** Andrey S. Gnedenkov, Alexey D. Nomerovskii, Aleksander K. Tsvetnikov, Sergey L. Sinebryukhov, Sergey V. Gnedenkov

**Affiliations:** Institute of Chemistry, Far Eastern Branch of the Russian Academy of Sciences, 159 Pr. 100-letiya Vladivostoka, 690022 Vladivostok, Russia

**Keywords:** steel, corrosion degradation, antifriction properties, electrochemical methods, cold spray technique, protective coating, composite material, polymer

## Abstract

Results of studying the properties of composite fluoropolymer-containing coatings formed by the cold spray (CS) method on the surface of constructional steel are presented. Different ways of protective coating formation are proposed. The composition of coatings was studied using SEM/EDX analysis. The incorporation of super-dispersed polytetrafluoroethylene (SPTFE) into the coating increases the corrosion resistance of the copper-zinc-based cold-sprayed coating. Analysis of the electrochemical properties obtained using EIS (electrochemical impedance spectroscopy) and PDP (potentiodynamic polarization) indicates that samples treated with SPTFE on a base copper-zinc coating showed lower corrosion current density and higher impedance modulus *(j*_c_ = 8.5 × 10^−7^ A cm^−2^, |*Z*|_f=0.1 Hz_ = 5.3 × 10^4^ Ω∙cm^2^) than the specimen with cold-sprayed SPTFE *(j*_c_ = 6.1 × 10^−6^ A cm^−2^, |*Z*|_f=0.1 Hz_ = 8.1 × 10^3^ Ω∙cm^2^). The best anticorrosion properties were revealed for the sample with a cold-sprayed base Cu-Zn layer annealed at 500 °C for 1 h, followed by SPTFE friction treatment and re-annealed at 350 °C for 1 h. The corrosion current density *j*_c_ of such a coating is 25 times lower than that for the base Cu-Zn coating. The antifriction properties and hydrophobicity of the formed layers are described. Obtained results indicate that cold-sprayed polymer-containing coatings effectively improve the corrosion and wear resistivity of the treated material.

## 1. Introduction

The good mechanical properties of mild steel have promoted its widespread use over the past few decades. The application of this type of steel has greatly contributed to the development of society due to its combination of good mechanical properties and low cost [1,2]. It is well known that the scope of low-alloy steel as one of the main structural materials largely depends on its mechanical properties [3].

However, the problem of corrosion failure of steel structures is still relevant. The industry constantly suffers significant losses as a result of corrosion, including degradation processes in reinforced concrete products [4]. The corrosion process affects the strength of the material and, accordingly, structures made from it, and products of degradation can cause failure of electrical equipment, plumbing, and heating systems [5]. In addition, there are indirect losses associated with the negative impact of corrosion products on the environment and a threat to safety [6].

Steel structures are actively used in all areas: railway communication, construction of buildings and structures, water supply, heat supply, and so on. A distinctive feature of steel is that it is often used in aggressive operating conditions (acid gases, aerosols of solutions of inorganic salts, dirt particles, temperature drops, UV radiation, static and dynamic loads [7,8,9]).

Currently, there are several ways to protect steel against corrosion, including the following: metal alloying with elements that are more resistant to participation in redox reactions (chromium, vanadium, nickel, manganese, and others); applying a protective coating; and change in the chemical composition of the environment. There are a number of works [10,11,12] related to the use of corrosion inhibitors in aggressive environments. For example, in [10] it was shown that the use of a “green” corrosion inhibitor made from the leaves of *Mangifera indica* L. in small quantities promotes the formation of a stable protective layer on the surface of steel, preventing the further process of metal degradation. In the work [12], the authors have shown a high efficiency of inhibitors (thiocarbohydrazide functionalized glucose derivatives) for carbon steel protection in simulated oilfield water.

However, the most rational, effective, and inexpensive method of protection is the application of coatings [13,14,15,16,17,18,19,20,21,22,23,24,25,26,27,28,29]. It is possible to use a paintwork material [13,19,30] (including those with additives of nanoparticles [25,31,32,33,34,35,36,37,38] and inhibitors [13,14,20,39,40,41,42,43,44,45,46,47]) and zinc coating to protect the material against its intensive destruction [48]. There is also a plasma electrolytic oxidation (PEO) method that allows one to create dense heterooxide coatings on valve metals (for example, Al, Mg, Ti, Nb, etc.) [49,50,51,52,53,54,55,56,57]. Unfortunately, this method is not practically applicable to steel due to the feature of the PEO process and the particulars of iron oxides. In this regard, another promising method proposed for applying a protective coating to steel is the cold spray (CS) [58,59,60].

Cold spray is a technology used to form, on the material surface, functional coatings with different thicknesses (10–100 μm) of various substances, mainly metals or composites [15,61]. This is a solid-state processing method in which micron-sized particles are accelerated (up to supersonic speeds) towards the substrate at relatively low temperatures (up to 600 K) [62]. When accelerating to supersonic speeds, the particles acquire high kinetic energy, and upon collision with the treated surface, both the particles and the substrate are deformed with the formation of a strong chemical and mechanical bond between the sprayed particles and the substrate [59]. Powder melting does not occur in this method. In contrast to the thermal spray method [63] and beam (laser or electronic) deposition methods, in the CS, the particles remain in a solid state (at least for the vast majority of metallic materials) throughout the entire process [64]. Due to the application of low temperatures, the coating obtained by the CS method has unique properties [15,61,65,66]. The microstructure and properties of the original powders are preserved, which avoids the formation of oxides or any other unfavorable structural changes, which increases the durability and effectiveness of the coatings. The adhesion of the substrate to the coating and the integrity of the protective layer are related to the plastic deformation of the particles upon impact. Coating adhesion can occur in the solid state of the deposited components without significant damage to the substrate, even for heat-sensitive materials. Although CS was originally used to deposit metals, it is also currently used to deposit polymers or ceramics [15,67]. Another way to enhance the mechanical and corrosion behavior of a wide range of steels is application of the cold spray technique with friction stir processing technology. The combination of these methods helps to improve the microstructure of the material by decreasing its porosity and number of voids [68,69,70].

The application of fluoropolymer materials as a coating component is found in [71,72,73]. In the work [71], fluoropolymer was incorporated into the pores of PEO coating on aluminum alloy. The formed layer effectively protected the material against corrosion. The use of super-dispersed polytetrafluoroethylene (SPTFE) is justified by the uniqueness of this material, which has a greater adhesion to the surface as compared to PTFE. SPTFE is characterized by a high content of the low molecular weight fraction of the fluoropolymer. This fluoropolymer was chosen because it provides hydrophobicity to the surface, which helps to reduce the intensity of the corrosion processes. In our previous work related to the formation of a composite PEO coating with fluoropolymer, the bonding strength of SPTFE to the substrate was studied [73]. It was revealed that for composite coatings, values of L_C2_ (the load at which the coating adhesion strength alteration began) are close to those of L_C3_ (the load at which scratching the coating down to the substrate occurred (plastic abrasion of the film to the metal)), which indicates a sufficient bonding strength of SPTFE to the substrate. In this work, SPTFE was applied to the metal surface using the cold spray technique for the first time.

In this article, the new composite polymer-containing coating consisting of metal matrix that was treated with polymer material was formed. A comparative analysis of the obtained copper-zinc and fluoropolymer protective coatings, formed by the cold spray technique, is carried out to protect mild steel St3. The morphological, electrochemical, and tribological characteristics of the coatings were studied. The effect of the application method (friction method or cold spray) of super-dispersed polytetrafluoroethylene (SPTFE) on the properties of the resulting protective coatings was also investigated. To the best of our knowledge, in the other studies that have dealt with the formation of cold-sprayed coatings, one can find only the results of mechanical tests [74,75,76,77]. Therefore, the data presented in the manuscript should help in improving the process of formation, the protective properties, and the efficiency of the CS composite layers.

Moreover, the problem of effective anticorrosion and antifriction protection of structural steel has not been completely solved yet in the world. The information presented in this work helps to improve the performance characteristics of the material, as well as to increase the efficiency of its application.

## 2. Materials and Methods

### 2.1. Preparing Samples

To study the morphological and electrochemical characteristics of the coatings, samples of St3 structural carbon steel (elemental composition in Table 1) with dimensions of 50 mm × 50 mm × 3 mm were prepared. Corundum powder grade K-00-04-02 (DIMET^®^) (powder particle size 200–250 µm) was used for cleaning and jet-abrasive preparation of the sample surface for metal coating. Powder grade C-01-11 (DIMET^®^) (powder particle size 5–50 µm) was used to apply a copper-zinc coating. The composition of the powder was as follows (wt. %): copper—38.3, zinc—28.6, corundum—33.1. The polymer layer was applied using a super-dispersed polytetrafluoroethylene powder (SPTFE, trademark FORUM^®^).

**Figure 1:**
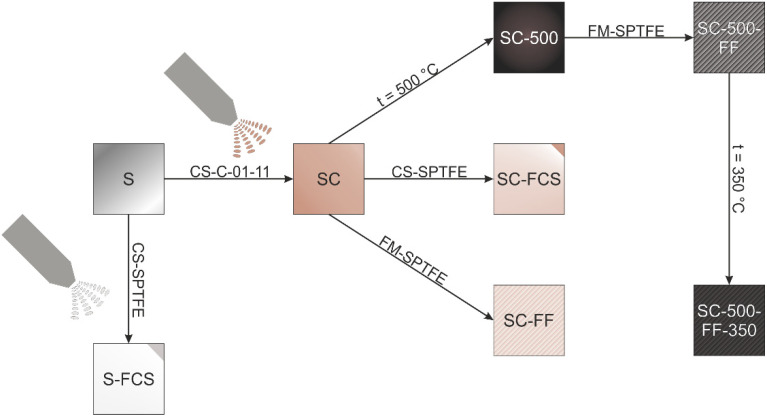
Scheme of sample preparation: CS—cold spray; FM—friction treatment; C-01-11—copper and zinc powder (DIMET^®^); SPTFE—super-dispersed polytetrafluoroethylene (FORUM^®^). Designation of samples is given in the text and Table 2.

The procedure for obtaining coatings is presented in Figure 1, where S is a sample of bare St3 steel without coating; S-FCS—sample S with SPTFE applied by cold spray; SC—St3 steel sample with cold-sprayed base copper-zinc coating; SC-FF—friction-treated with SPTFE sample SC; SC-FCS—sample SC with SPTFE applied by cold spray; SC-500—SC sample annealed at 500 °C for 1 h; SC-500-FF—friction treated with SPTFE sample SC-500; SC-500-FF-350—sample SC-500-FF re-annealed at 350 °C for 1 h. Information about the types of sample treatment is summarized in Table 2.

**Figure 2:**
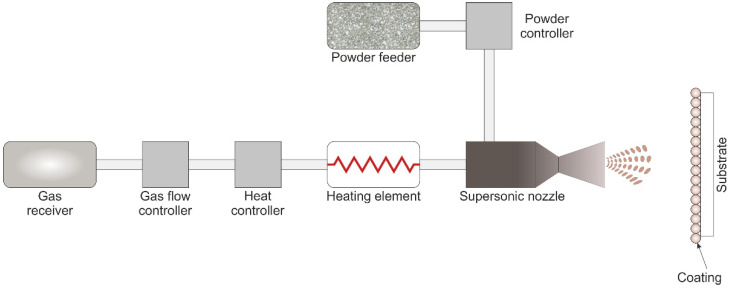
Scheme of the cold spray DIMET^®^ equipment.

The deposition of materials by the CS method was carried out using DIMET^®^ equipment, as shown in Figure 2, using compressed air at a pressure of 5 bar, without preheating. Round nozzle with a diameter of 5 mm was used. The distance from the nozzle to the substrate surface was equal to ~4 cm. The nozzle was moved relative to the substrate at a speed of ~1 cm/s. To apply SPTFE by the friction method, a plastic brush with PVC bristles ~200 µm in diameter was used. Thermal treatment of the samples was carried out in a muffle furnace L 9/13/B180 (Nabertherm, Lilienthal, Germany).

The thickness of the formed coatings was controlled using a model 908.750 micrometer (Schut Geometrical Metrology, Groningen, Germany). Weight control of the samples was performed using an analytical balance AUW120D (Shimadzu, Kyoto, Japan).

### 2.2. Analysis of Coatings

The surface morphology of the samples was studied using an EVO 40 scanning electron microscope (Carl Zeiss, Jena, Germany) equipped with an INCA X-act instrument module (Oxford Instruments, Oxford, UK) for elemental analysis by means of energy-dispersive X-ray spectroscopy (EDX).

Electrochemical studies were carried out using potentiodynamic polarization and electrochemical impedance spectroscopy using a Modulab XM MTS electrochemical system (Solartron Analytical, Oak Ridge, TN, USA). The tests were carried out at room temperature in a three-electrode cell in a 3.5 wt. % NaCl solution. The area of the investigated surface was 1 cm^2^. The platinized niobium mesh served as a counter electrode, and the silver chloride (Ag/AgCl) electrode (potential versus a normal hydrogen electrode is equal to 0.197 V) served as a reference electrode. Before electrochemical measurements, the samples were kept in the electrolyte for 15 min to stabilize the electrode potential. The frequency value during electrochemical impedance spectroscopy varied in the range from 100 kHz to 10 mHz with a logarithmic sweep of 10 points per decade. Potentiodynamic measurements were carried out at a sweep rate of 1 mV/s. The sample was polarized in the anodic direction in the potential range from *E*_c_ − 0.2 V up to *E*_c_ + 0.7 V. The corrosion potential, *E*_c_, and the corrosion current density, *j*_c_, were determined from the intersection of the extrapolated anodic and cathodic Tafel segments of the polarization curve. The polarization resistance (*R*_p_) was calculated in a separate experiment using a linear polarization resistance test in accordance with Equation (1) [78]. The specimens were polarized from (*E*_c_ − 0.02) V to (*E*_c_ + 0.02) V. The scan rate was equal to 0.167 mV s^−1^.

*R*_p_ = ∆*E*/∆*I* (1)

The tribological properties of composite coatings were studied on a Tribometer TRB-S-DE (CSM Instruments, Peseux, Switzerland) (rotation mode with a sliding speed of 50 mm/s and a normal load of 15 N). The 10 mm corundum ball (α-Al_2_O_3_) was used as a counterbody. The wear track diameter was equal to 10 mm. To estimate the wear, the profiles were studied after the tests, using a Surtronic 25 profilometer (Taylor Hobson, Leicester, UK). The wear (*P*, mm^3^/(N × m)) was estimated using Equation (2):

*P* = ∆*V*/*NF*, (2)

where ∆*V* is the sample volume loss during the test, *N* is the distance, and *F* is the applied normal load. The sample volume loss was calculated according to ∆*V* = *S∙l*, where *l* is the length of the track, and *S* is the area of the cross-section.

The wettability of the formed coatings was studied by the sessile drop method on a DSA100 drop shape analyzer (KRÜSS, Hamburg, Germany). The latter comprises an optical contact angle (CA) technique. This technique is used to estimate the wetting properties of the localized area on a solid surface. According to this method, the angle between the baseline of the drop and the tangent at the three-phase boundary was measured. The test liquid was distilled water. The volume of the drop was 10 μL. For calculations of CA, the Young–Laplace method, taking into account the gravitational distortions of liquid drop form, was used. At least five measurements were carried out to obtain contact angle values.

**Figure 3:**
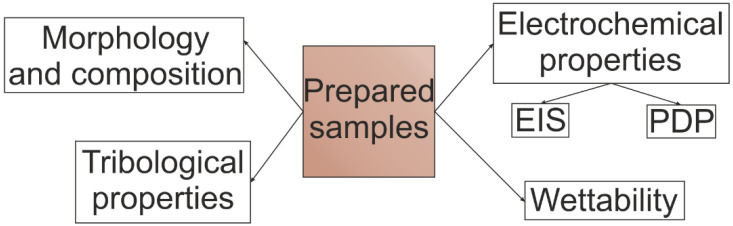
Scheme of the used analysis of samples.

The scheme of the performed tests is shown in Figure 3.

## 3. Results and Discussion

### 3.1. Morphology Analysis

**Figure 4:**
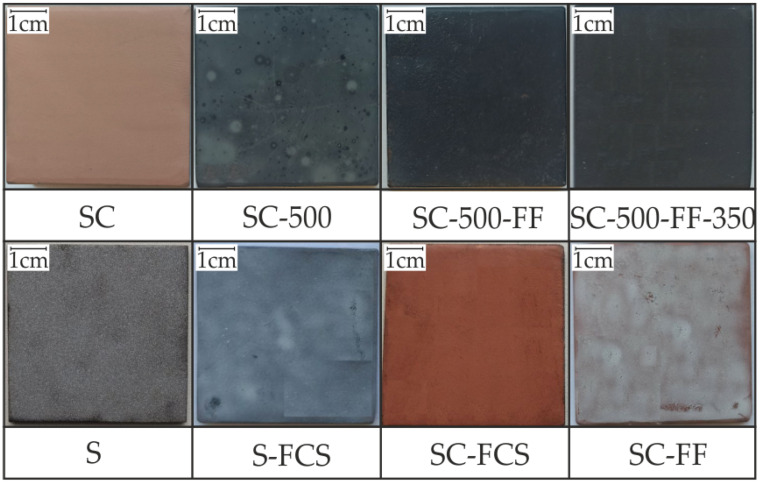
Images of the samples under study.

**Figure 5:**
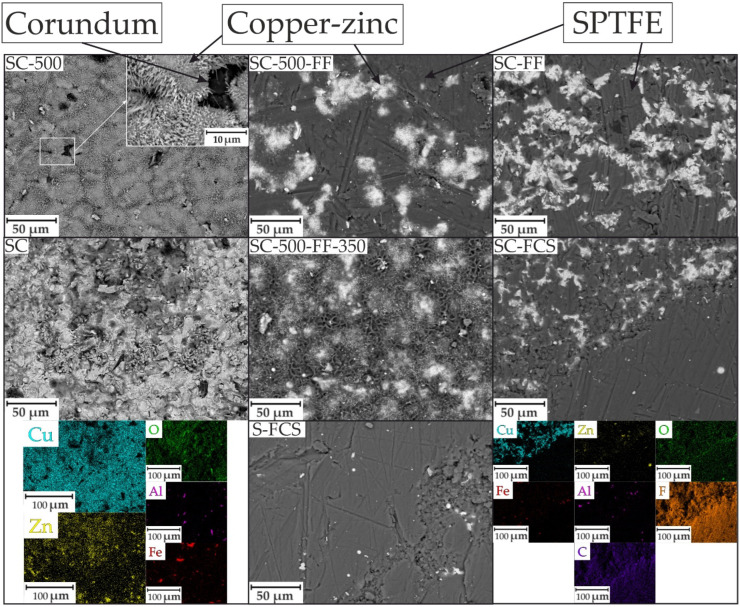
SEM images of the surfaces of coatings formed on St3 steel samples and element distribution maps of SC and SC-FCS samples.

The appearance of the test samples is shown in Figure 4. The morphologies of the various coatings formed using the CS method are depicted in Figure 5. The coating with a rough surface was formed by applying a copper-zinc powder by cold spray on a steel substrate (Figure 5, SC). EDX maps of the element distribution of the SC sample showed a uniform distribution of copper and zinc (as the main components of the cold-sprayed material) in the coating structure. Oxygen is also present on the surface, which is associated with partial oxidation of the powder. In some regions, the presence of iron (the main component of steel) is visible. This is related to a feature of the cold spray process where partial erosion of the substrate surface occurs to fix the powder particles, and not to the incomplete filling of the sample surface with copper and zinc powder. Inclusions of aluminum oxide were determined (this component is present in powder C-01-11). The coating thickness was about 30–40 µm.

After heat treatment at 500 °C for 1 h, the oxide microstructure in the form of needle-shaped crystallites is formed on the coating (Figure 5, SC-500). The formation of such a structure significantly increases the surface roughness compared to the base SC coating. This morphological feature indicates the possibility of further functionalization of the coating, which will change the properties of the surface layer.

SEM images of the surface of SC-FF and SC-500-FF samples obtained after SPTFE friction treatment of SC and SC-500, respectively, are shown in Figure 4. Dark areas of the specimen correspond to the zones containing SPTFE, and light areas to the base copper-zinc coating. An analysis of the results indicates a higher continuity of the SPTFE layer on the surface of the SC-500-FF sample compared to SC-FF, which is a consequence of the positive effect of the temperature treatment of the SC sample on the surface modification with the polymer.

Further heat treatment of the SC-500-FF sample at 350 °C results in the removal of the volatile SPTFE fraction, which can cause a change in the protective properties of the formed layer.

It should be noted that the cold spray of SPTFE (Figure 5, SC-FCS, S-FCS) significantly covers the metal surface compared to the friction method of applying the polymer (Figure 5, SC-FF). Maps of the element distribution of the SC-FCS sample (Figure 5, SC-FCS) showed a copper-zinc site and SPTFE site on the surface. This indicates the functionalization of the copper-zinc base coating with fluoropolymer powder.

**Figure 6:**
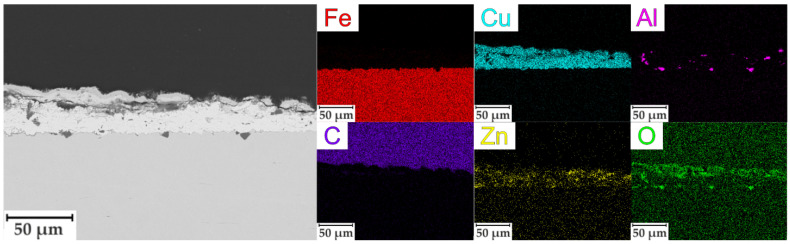
SEM image of the cross-section and EDX element distribution maps of SC-500 sample.

The SEM image of the cross-section and maps of the element distribution of the sample SC-500 are presented in Figure 6. The SEM image shows that the surface layer is represented by needle-shaped crystallites. The coating is dense, and the particles of copper and zinc are evenly distributed in the coating. There are also particles of corundum in the coating composition. An oxide layer was formed on the surface of the coating, as evidenced by the distribution of oxygen.

### 3.2. Electrochemical Properties

#### 3.2.1. Electrochemical Impedance Spectroscopy Studies

**Figure 7:**
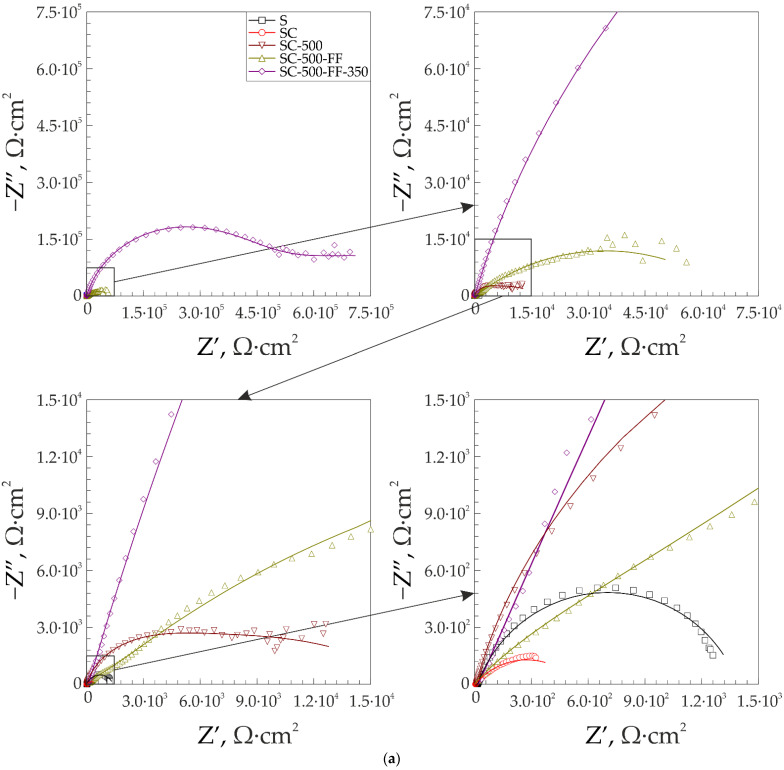

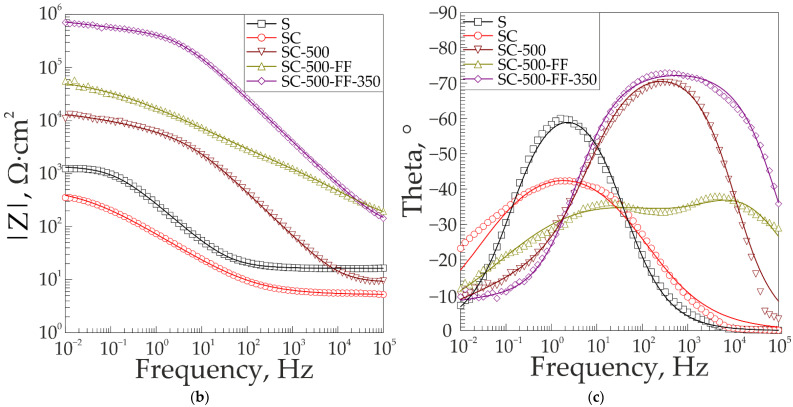
Nyquist (**a**) and Bode plots (**b**,**c**) for the samples at various stages of processing.

**Figure 8:**
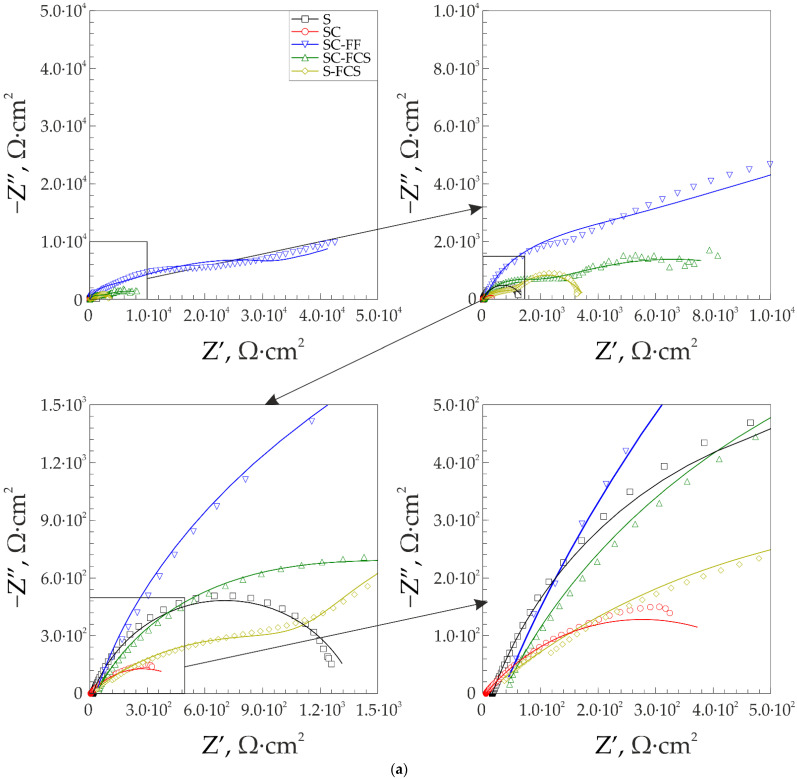

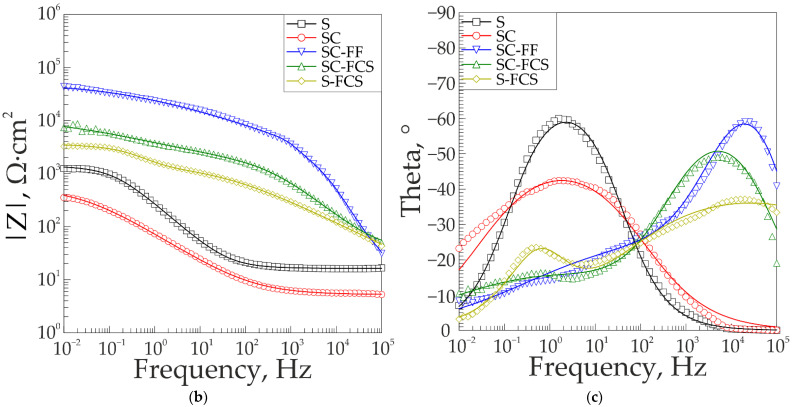
Nyquist (**a**) and Bode plots (**b**,**c**) for the samples with the different means of SPTFE deposition.

**Figure 9:**
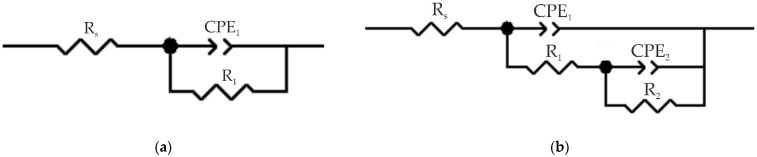
Equivalent electrical circuits used to fit the experimental impedance spectra of coatings S and SC (**a**) and SC-500, SC-500-FF, SC-500-FF-350, SC-FCS, SC-FF, and S-FCS (**b**).

The results of the impedance spectroscopy are shown in Figure 7 and Figure 8. The spectra of samples S and SC are described by an equivalent electrical circuit (EEC) using a single R-CPE element (Figure 9a). The spectra of samples SC-500, SC-500-FF, SC-500-FF-350, SC-FCS, SC-FF, and S-FCS are described by an equivalent electrical circuit using two series-parallel connected R-CPE chains (Figure 9b). The calculated parameters of all EEC elements are presented in Table 3. Samples S and SC (R_1_-CPE_1_) correspond to a layer of natural oxide film on the steel surface (sample S) and a layer of copper-zinc coating (sample SC). After annealing the SC sample at 500 °C for 1 h, an oxide layer is formed on the surface of the coating (Figure 5, SEM image of SC-500). To describe the impedance spectrum of this sample, two series-parallel connected R-CPE elements were used, where R_1_-CPE_1_ corresponds to the oxide layer of the copper-zinc coating, and R_2_-CPE_2_ describes the base Cu-Zn coating. 

It should be noted that all spectra of samples with different SPTFE deposition can be described by an EEC consisting of two series-parallel connected R-CPE chains, where R_1_-CPE_1_ corresponds to the part of the coating containing SPTFE layer, and R_2_-CPE_2_ describes the other part of the coating without polymer. For example, to describe the spectrum of sample SC-500-FF, two series-parallel connected R-CPE chains were used, where R_1_-CPE_1_ corresponds to the outer part of the coating that is mostly covered with SPTFE, and R_2_-CPE_2_ describes the inner part of the protective layer. A similar description of the spectrum is proposed for SC-500-FF-350, since the difference from the previous sample lies in the partial evaporation and melting of SPTFE and, accordingly, its deep impregnation due to heat treatment. Table 3 includes R_1_, R_2_ and CPE_1_, CPE_2_ (n and Q are the exponential coefficient and CPE constant, respectively), which describe the resistive and capacitive character of the abovementioned layer, respectively. R_s_ is the solution resistance, and χ**^2^** is chi-square value.

According to electrochemical impedance spectroscopy data, thermal treatment of the sample at 500 °C (SC-500 sample) forms an additional barrier layer on the surface. The impedance modulus at a frequency of 0.1 Hz increased nine times compared to the SC sample (|*Z*|_f=0.1 Hz_ = 1.17 × 10^3^ Ω∙cm^2^ for the SC sample and |*Z*|_f=0.1 Hz_ = 1.05 × 10^4^ Ω∙cm^2^ for the SC-500 sample). 

**Figure 10:**
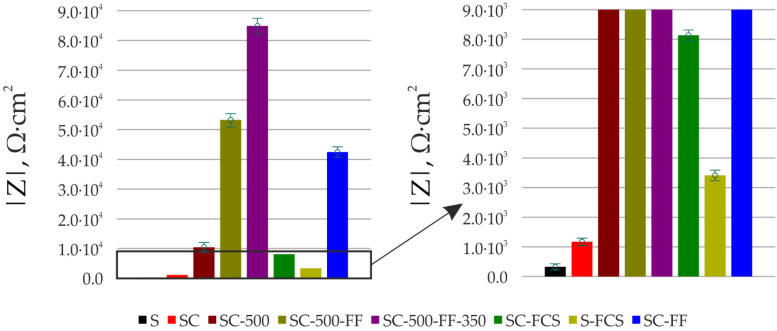
Evolution of impedance modulus measured at a frequency of 0.1 Hz for sample with different coatings.

Further modification of the coating using SPTFE treatment increases its protective properties without changes in the equivalent electrical circuit. This is due to the fixation of SPTFE at the microdefect sites in the coating (Figure 5, SC-500 and SC-500-FF). Samples with SC-500-FF and SC-FF coating have comparable values of the impedance modulus at the lowest frequency, |*Z*|_f=0.1 Hz_, namely 5.33 × 10^4^ Ω∙cm^2^ and 4.25 × 10^4^ Ω∙cm^2^, respectively, which indicates similar characteristics of the protective properties of these layers and a higher corrosion resistance of the SC-FF coating as compared to the SC-500 (|*Z*|_f=0.1 Hz_ = 1.05 × 10^4^ Ω∙cm^2^). Further heat treatment of the sample SC-500-FF at 350 °C (SC-500-FF-350) improves the protective properties of the resulting coating, due to the SPTFE partial melting and penetration to the inner part of surface layer that provides the closing defects with polymer (|*Z*|_f=0.1 Hz_ = 8.49 × 10^4^ Ω∙cm^2^). Deposition of SPTFE on bare steel by cold spray showed lower impedance modulus at a frequency of 0.1 Hz than on steel with copper-zinc coating (|*Z*|_f=0.1 Hz_ = 3.41 × 10^3^ Ω∙cm^2^ for the S-FCS sample and |*Z*|_f=0.1 Hz_ =8.14 × 10^3^ Ω∙cm^2^ for the SC-FCS sample). The impedance modulus measured at 0.1 Hz for all coatings, depending on the St3 surface treatment, is shown in Figure 10. The analysis of the calculated parameters of the EEC elements showed the changes in the surface morphology (changing the thickness and integrity of the protective layer according to Q_1_ and Q_2_ evolution) and the resistance of the formed coatings due to the cold spray, oxide layer formation, and polymer treatment (Table 3).

#### 3.2.2. Potentiodynamic Polarization Measurements

**Figure 11:**
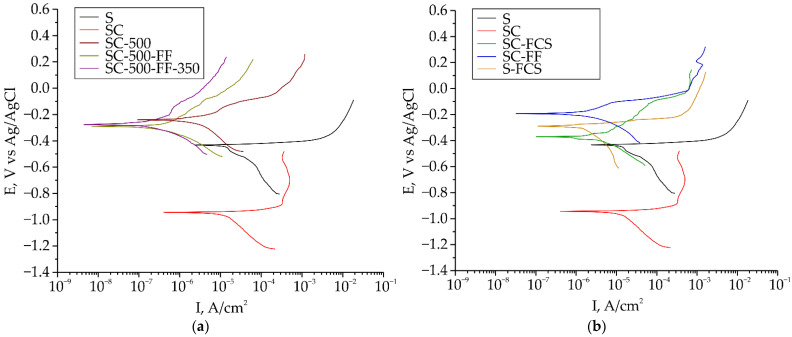
Polarization curves for the samples at various stages of processing (**a**) and for the samples at different types of SPTFE deposition (**b**).

The shift of the corrosion potential to the more negative value for the sample SC as compared to sample S from −436.8 mV to −946.1 mV (Table 4) was established. This is due to the presence of zinc in the composition of the coating. Zn, as a more electrochemically active metal, will play the role of protector and the first of the coating components to corrode, protecting the substrate material and structure as a whole. Therefore, there is also a slight decrease in the corrosion current density from 14.2 µA∙cm^−2^ for sample S to 11.5 µA∙cm^−2^ for sample SC (Figure 11). Further surface treatment (thermal action and formation of a SPTFE layer) contributes to an increase in the corrosion potential value to –203.5 mV and a decrease in the corrosion current density down to 0.85 µA∙cm^−2^ (Figure 11a, SC-500-FF). Thermal treatment of SC-500-FF at 350 °C improved the corrosion resistance of the coating: the corrosion current decreased down to 0.52 µA∙cm^−2^.

Cold spray treatment using SPTFE of the base copper-zinc coating and bare steel changes the corrosion potential to the more noble value and reduces the corrosion current by an order of magnitude (Figure 11b).

### 3.3. Tribological Properties

**Figure 12:**
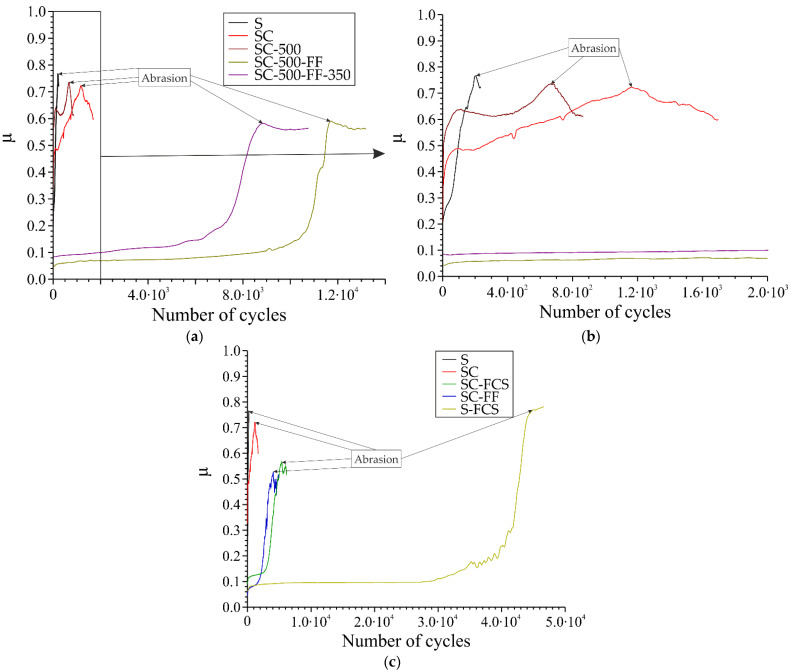
The results of tribological tests (dependence of friction coefficient on number of cycles) for the samples at various stages of processing (**a**,**b**) and at different types of SPTFE deposition (**c**). Samples S and SC are shown for comparison.

Tribological tests of the investigated samples (Figure 12, Table 5) revealed some differences in the behavior of coatings obtained by various methods. The moment of the coating abrasion to the metal is marked with an arrow. Sample S withstood 2.0∙10^2^ cycles until the maximum value of the coefficient of friction was reached (μ = 0.77); the surface was protected by a natural oxide film. Copper-zinc coating (SC sample) withstood 1.2 × 10^3^ rotation cycles until μ = 0.72. However, after heat treatment, this coating could only withstand 6.6 × 10^2^ cycles, which was associated with diffusion and chemical processes occurring in the coating during heat treatment. Treatment using the SPTFE friction method increased abrasion resistance up to 1.2 × 10^4^ cycles. Post heat treatment decreased the amount of SPTFE in the coating and, therefore, the number of cycles also decreased down to 8.7 × 10^3^.

Samples with the SPTFE layer coated on the surface of copper-zinc using both the friction method and cold spray method show approximately the same number of cycles up to coating wear: 4.4 × 10^3^ and 5.4 × 10^3^, respectively. The maximum number of rotation cycles withstood by a sample was that of bare steel with SPTFE coated by the cold spray method.

### 3.4. Wettability of Coatings

Analysis of the wettability data indicates that all coatings are hydrophobic (Table 6). However, the hydrophobicity of the specimens is not only a result of the chemical nature of SPTFE. Comparing the contact angles for the SC-FF and SC-FCS samples enables one to conclude that the developed relief of the surface is a very important factor for hydrophobicity, since SPTFE coated by friction deposition forms a smooth layer on the surface, while polymer particles deposited by cold spray remain on the surface in their original form and provide more convoluted relief. Perhaps, the higher contact angle for SC-FCS caused the multimodal roughness of this sample (this specimen has the highest measured value of R_a_—arithmetical mean deviation of the profile). The contact angle of SC-500 is similar to that of S-FCS. However, the microlevel roughness described by the parameter of R_a_ cannot completely reveal the reason for the wettability change (Table 6). The multimodal roughness includes the microscale and nanoscale morphology features of the surface. Therefore, the evolution of the contact angle is a result of the nanoscale roughness effect of the investigated surface. 

### 3.5. Mechanism of the Protective Effect of the Composite Coating

Protective layer formation using the cold spray technique makes it possible to preserve the original qualitative composition of the sprayed powder in the coating. The formed CS coating showed good wear resistance (sample SC, 1.2 × 10^3^ cycles), which was 10 times higher than unprotected steel (sample S, 2.0 × 10^2^ cycles). Undoubtedly, SPTFE plays a significant role in the formation of more stable and corrosion-resistant coating (impedance modulus at a frequency of 0.1 Hz for SC-500-FF-350 |*Z*|_f=0.1 Hz_ = 8.49 × 10^4^ Ω∙cm^2^, increased by 73 times as compared to SC sample |*Z*|_f=0.1 Hz_ = 1.17 × 10^3^ Ω∙cm^2^). It was established that base copper-zinc coating, due to the developed surface, makes it easier for SPTFE particles to attach to the surface. SPTFE also increases the wear resistance of protective coating (sample SC-500-FF, 1.2 × 10^4^ cycles).

Despite the good electrochemical properties of some coatings, their wear resistance indicates the limited application of such coatings. Summarized information regarding the corrosion properties and wear resistance of the formed coatings is presented in Table 7. Two of the best coatings are: SC-500-FF-350 (S with cold-sprayed base copper-zinc coating annealed at 500 °C for 1 h with friction treated with SPTFE re-annealed at 350 °C for 1 h) and S-FCS (S with SPTFE applied by cold spray). 

**Figure 13:**
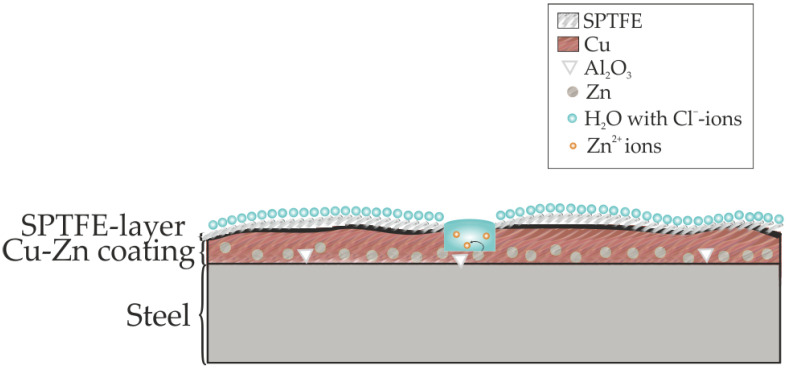
The proposed mechanism of the protective action of the composite coating obtained using the cold spray method.

In accordance with the obtained data, the following scheme (Figure 13) was proposed for the description of the protective effect of the coating containing SPTFE applied by cold spray. SPTFE creates a barrier layer that prevents the penetration of water with aggressive ions to the deeper layers of the coating and to the substrate. In the event of defect formation on the surface of the coating, zinc will play the role of protector, dissolving first. After the dissolution of all the zinc, the iron substrate will begin to corrode. 

The obtained data promote more efficient use of mild steel by means of the composite coatings with a new composition formed using the cold spray technique. Moreover, this study provides information additional to the other works, where only the mechanical properties of CS coatings were investigated [74,75,76,77]. Therefore, the significance of this work regarding expansion of the practical applications of this structural material in corrosive media was highlighted. 

## 4. Conclusions

The results obtained in this study enable one to conclude the following:

− The composite fluoropolymer-containing protective coatings were formed on the surface of St3 low carbon steel using the cold spray method;
− The treatment of the base copper-zinc CS-layer with super-dispersed polytetrafluoroethylene increases the corrosion and wear resistance of the material (according to electrochemical and tribological tests);
− According to the performed analysis, the best protective properties were registered for the SC-500-FF-350 sample with cold-sprayed base copper-zinc coating annealed at 500 °C for 1 h, friction-treated with SPTFE, and re-annealed at 350 °C for 1 h, and the S-FCS sample with SPTFE applied by cold spray;
− The multi-stage treated coating is not inferior to the one with cold-sprayed SPTFE in terms of the combination of protective properties;
− The obtained data promote more efficient use of mild steel by means of the composite coatings with a new composition intended to expand the practical applications of this structural material in corrosive media.

## Figures and Tables

**Table 1:** Elemental composition of St3 steel.

Element	C	Si	Mn	Ni	S	P	Cr	N	Cu	As	Fe
wt. %	0.14–0.22	0.15–0.3	0.4–0.65	<0.3	<0.05	<0.04	<0.3	<0.008	<0.3	<0.08	balance

**Table 2:** Designation of the sample with different coatings formed on St3 steel.

Sample	Type of the Treatment
S	Without coating
SC	S with cold-sprayed base copper-zinc coating
SC-500	SC annealed at 500 °C for 1 h
SC-500-FF	SC-500 friction-treated with SPTFE
SC-500-FF-350	SC-500-FF re-annealed at 350 °C for 1 h
SC-FF	SC friction-treated with SPTFE
SC-FCS	SC with SPTFE applied by cold spray
S-FCS	S with SPTFE applied by cold spray

**Table 3:** Calculated EEC element parameters for samples with different coatings.

Sample	R_s_ (Ω cm^2^)	CPE_1_		R_1_ (Ω cm^2^)	CPE_2_		R_2_ (Ω cm^2^)	χ*^2^*
		Q_1_ (S cm^2^ s^n^)	n		Q_2_ (S cm^2^ s^n^)	n		
S	33	8.4 × 10^−4^	0.78	1.4 × 10^3^				4.1 × 10^−4^
SC	28	4.8 × 10^−3^	0.56	5.4 × 10^2^				1.0 × 10^−3^
SC-500	29	9.9 × 10^−6^	0.84	3.8 × 10^3^	1.1 × 10^−4^	0.38	1.4 × 10^4^	1.1 × 10^−3^
SC-500-FF	31	2.1 × 10^−6^	0.63	1.2 × 10^3^	2.0 × 10^−5^	0.43	6.5 × 10^4^	7.2 × 10^−4^
SC-500-FF-350	32	1.9 × 10^−7^	0.82	4.1 × 10^5^	4.8 × 10^−6^	0.35	7.1 × 10^5^	3.6 × 10^−4^
SC-FF	30	5.3 × 10^−8^	0.87	2.1 × 10^3^	1.4 × 10^−5^	0.34	4.9 × 10^4^	1.0 × 10^−3^
SC-FCS	30	2.3 × 10^−6^	0.72	1.5 × 10^3^	1.7 × 10^−4^	0.33	1.0 × 10^4^	1.0 × 10^−3^
S-FCS	33	6.3 × 10^−5^	0.44	1.5 × 10^3^	2.3 × 10^−4^	0.84	2.1 × 10^3^	5.6 × 10^−4^

**Table 4:** Calculated Tafel parameters for samples with different coatings.

Sample	*β*_a_ (mV/Decade)	*−β*_c_ (mV/Decade)	*E*_c_ (mV vs. Ag/AgCl)	*j*_c_ (A cm^−2^)	*R*_p_ (Ω cm^2^)
S	51.3	284.2	−436.9	1.4 × 10^−5^	6.8 × 10^3^
SC	22.3	264.2	−946.1	1.2 × 10^−5^	2.5 × 10^3^
SC-500	128.3	327.9	−240.1	3.7 × 10^−6^	1.3 × 10^4^
SC-500-FF	346.9	211.7	−294.2	8.5 × 10^−7^	4.1 × 10^4^
SC-500-FF-350	519.2	226.7	−273.1	5.2 × 10^−7^	9.8 × 10^4^
SC-FF	288.5	218.9	−196.3	4.5 × 10^−6^	1.2 × 10^4^
SC-FCS	233.5	241.2	−372.3	6.1 × 10^−6^	6.5 × 10^3^
S-FCS	33.6	239.9	−286.9	1.4 × 10^−6^	8.0 × 10^3^

**Table 5:** Tribological tests of the specimens with different types of coatings.

Sample	Number of Cycles	Wear (mm^3^ N^−1^ m^−1^)
S	2.0 × 10^2^	8.3 × 10^−4^
SC	1.2 × 10^3^	5.4 × 10^−4^
SC-500	6.6 × 10^2^	6.8 × 10^−4^
SC-500-FF	1.2 × 10^4^	9.0 × 10^−5^
SC-500-FF-350	8.7 × 10^3^	2.1 × 10^−4^
SC-FF	4.4 × 10^3^	9.3 × 10^−5^
SC-FCS	5.4 × 10^3^	2.9 × 10^−4^
S-FCS	4.1 × 10^4^	5.1 × 10^−5^

**Table 6:** Wettability and roughness parameters of the specimens with different types of coatings.

Sample	Contact Angle (°)	R_a_, μm
S	97.7	0.7
SC	135.9	3.0
SC-500	144.1	1.6
SC-500-FF	118.7	1.5
SC-500-FF-350	140.2	1.7
SC-FF	112.1	2.9
SC-FCS	158.8	3.1
S-FCS	154.2	1.1

**Table 7:** Results of various coating tests (where 1—the best, 7—the worst; the smaller the number, the better protective properties). Estimated parameter for columns: EIS—the impedance modulus at a frequency of 0.1 Hz, PDP—the corrosion current density, wear—the number of cycles to the coating wear, wettability—the contact angle, total—summarized score (the smaller the score, the better protective properties). The samples with the best protective coatings are marked with green color.

Sample	EIS	PDP	Wear	Wettability	Total
SC	7	7	6	5	25
SC-500	4	4	7	3	18
SC-500-FF	2	2	2	6	12
SC-500-FF-350	1	1	3	4	9
SC-FF	5	5	5	7	22
SC-FCS	6	6	4	1	17
S-FCS	3	3	1	2	9

## Data Availability

Not applicable.
